# High-Resolution Epitope Mapping and Affinity Binding Analysis Comparing a New Anti-Human LAG3 Rabbit Antibody Clone to the Commonly Used Mouse 17B4 Clone

**DOI:** 10.3390/antib11040060

**Published:** 2022-09-21

**Authors:** P. Daniel Warren, Mark S. Dodson, Margaret H. Smith, Terry H. Landowski, John Douglas Palting, Penny Towne

**Affiliations:** Roche Tissue Diagnostics Ventana, Tucson, AZ 85755, USA

**Keywords:** LAG3, rabbit SP464 clone, mouse 17B4 clone, epitope mapping, capillary gel electrophoresis, surface plasmon resonance, immunohistochemistry

## Abstract

Lymphocyte activation gene 3 (LAG3) is a T cell inhibitory receptor that promotes tumor cell immune escape and is a potential target for cancer diagnostic and immunotherapeutic applications. We used automated capillary electrophoresis (ACE), surface plasmon resonance (SPR), and immunohistochemistry (IHC) to compare the binding characteristics of a new anti-LAG3 rabbit antibody clone, SP464, with the thirty-year old and extensively used anti-LAG3 mouse 17B4 clone. The rabbit SP464 clone exhibited between 20× to 30× greater binding to LAG3 than did the mouse 17B4 clone. Using these tools, we precisely mapped the relative locations of the epitopes of these two antibodies. The SP464 and 17B4 minimal epitopes were localized to separate, but overlapping, sub-fragments within the amino-terminal fifteen acids of the original thirty-mer peptide immunogen used to generate both antibodies. Application of this approach for quantifying the effects of alanine substitutions along the minimal SP464 epitope identified two amino acids essential for binding and four amino acids that likely contribute towards binding. Together, ACE, SPR, and IHC constitute a powerful orthologous approach for comparing antibody-binding characteristics and for fine mapping of linear epitopes within short immunogens. Our results indicate that the rabbit clone SP464 may be useful for assessing LAG3 expression.

## 1. Introduction

The immunomodulatory protein, Lymphocyte activation gene 3 (LAG3) (CD223) was discovered over 30 years ago by Triebel et al. [1] as a novel member of the immunoglobulin (Ig) superfamily. LAG3 is a transmembrane protein with four extracellular Ig-like domains that show approximately 20% amino acid homology with CD4. Surface expression of the LAG3 receptor is induced on CD4+ and CD8+ T cells upon antigen stimulation, and functions as a negative regulator of T cell cytotoxicity. Recent studies have shown that the inhibitory function of LAG3 strongly correlates with the level of surface expression and subsequently, high LAG3, on tumor infiltrating lymphocytes, is associated with a poor prognosis [2,3]. Preclinical and early clinical trials have indicated that active LAG3 promotes tumor cell immune escape. Inhibition of LAG3 restores T cell activity, and is synergistic with inhibition of PD-1, making it a prime target for immunotherapy. The recently completed RELATIVITY-047 trial (Clinicaltrials.gov NCT0340922) demonstrated a significant improvement in median progression-free survival in patients with metastatic melanoma treated with a combination of anti-LAG3 BMS 986016 (Relatlimab), and anti-PD-1 (Nivolumab) compared to anti PD-1 alone [4]. This study led to FDA approval of a combination checkpoint inhibitor therapy for previously untreatable metastatic or unresectable melanomas. The emergence of LAG3 targeted therapies and co-development of immunotherapy with companion diagnostic (CDx) assays has established a need for new and well-characterized anti-LAG3 in vitro diagnostic reagents (IVDR) [5].

Immunohistochemistry (IHC) is a well-established technology for clinical diagnosis and CDx assays that is inexpensive and widely available; however, the utility of IHC is wholly dependent on reliable IVDR antibodies. The characterization and development of clinically useful antibodies faces a variety of persistent technical difficulties. In particular, identifying the precise location of an antibody binding site within a larger epitope is a critical step towards determining the specificity of an antibody for its target. A number of factors associated with identifying the actual binding sequence and characterizing binding with its cognate antibody are well documented [6]. Characterization studies include both physical (e.g., thermodynamic) and biological (e.g., site-directed mutation) analyses for characterizing antibody-protein interactions. Such studies require a number of tools, such as biosensor-based technologies, either surface plasmon resonance (SPR) [7,8] or bio-layer interferometry [9,10], ELISA [11], X-ray crystallography [12,13], nuclear magnetic resonance [14,15], mass spectroscopy [16,17], and bioinformatics aided by in silico technologies [18]. These techniques, when coupled with synthetic linear peptides, enables even more advancement in the area of fine epitope mapping techniques [19]. Efficiency improvements upon the ‘Geysen pepscan’ [20] and resulting multitude of techniques [21] have further expanded availability of these tools. However, fine epitope mapping has also identified some shortcomings of these tools when applied in a vacuum [14,22,23]. 

Currently, the most extensively used LAG3 specific antibody is the mouse anti-LAG3 monoclonal 17B4, which was developed thirty years ago [24]. Herein, we introduce a new rabbit-based clone, SP464, elicited from the same 30-mer immunogen as the 17B4 mouse clone and explore key binding differences between the clones. Additionally, we demonstrate the application of immunogen fragmentation and alanine-scanning mutagenesis to fine-map the minimum epitope sequences of both clones using the orthogonal technologies of Western blot, automated capillary electrophoresis (ACE), SPR, and IHC. The work presented here demonstrates the benefits of adapting this combinatorial approach as a standard method for development of an antibody as an IVD CDx assay.

## 2. Materials and Methods

### 2.1. Antibodies, Peptides and Proteins

The Roche Tissue Diagnostics VENTANA LAG3 rabbit (SP464) and mouse LAG3 (17B4) antibodies were from Spring Biosciences Corporation. Anti-FAPα antibody (SP325) and anti-CD8 alpha antibody (SP239) were obtained from Abcam. Peptides were synthesized by Anaspec, Freemont, CA. The lyophilized peptides were dissolved in dH2O, adjusted to a concentration of 2 mM, and stored at −80 °C until use. The recombinant LAG3 protein was from ABCAM.

### 2.2. Cell Lysates

Human lymphoma (HH, CRL-2105) cell line was from the ATCC. The human Hodgkin lymphoma (L-428, ACC 197) cell line was from the DSMZ. Human ataxia-telangiectasia mutated gene B lymphocyte cell line (AT, GM05126) was from the Coriell Institute. All cell lines were authenticated by the vendor, were maintained in a humidified incubator (37 °C, 5% CO_2_ and 95% air), and used at less than 10 passages from origin. Cells were propagated in DMEM & RPMI 1640 medium, respectively, supplemented with 10% heat-inactivated FBS and 1% penicillin/streptomycin at 37 °C with 5% CO_2_. Cells were cultured to the desired density (1 × 10^7^ mL) and pelleted by centrifugation at 100× *g* at room temperature (RT) for 3 min. Pellets were washed with cold DPBS followed by 1× protease and phosphatase inhibitor solution in cold DPBS, and pelleted by centrifugation at 16,000× *g* at 4 °C for 20 min. Cell pellets were then lysed in RIPA lysis buffer supplemented with a protease and phosphatase inhibitor cocktail. Debris was removed by centrifugation at 15,000× *g* at 4 °C for 30 min, and the cleared lysate was stored at 4 °C.

### 2.3. Formalin Fixed Cell Line Blocks

Cells were propagated, harvested, and pelleted as described above. The cell pellets were suspended in phosphate buffered saline containing one percent low melt agarose. The agarose cell blocks were then formalin fixed and embedded in paraffin.

### 2.4. Tissue

Formalin fixed paraffin embedded (FFPE) tissues were commercially sourced and de-identified for use in research studies (US Biolabs). 

### 2.5. Western Blot Reagents and Apparatus

NuPAGE MOPS 20× Running Buffer, NuPAGE 20× Transfer Buffer, NuPAGE 4–12% Bis-Tris Mini Gels, iBright molecular weight standards, anti-rabbit and mouse WesternBreeze Chemiluminescent Kits, Invitrolon PVDF 0.20 µm membranes, XCell SureLock Mini Cell, iBLOT2 Module, and a PowerEase 500 Power Supply were from Life Technologies.

### 2.6. Automated Capillary Electrophoresis (ACE) Reagents and Instrumentation

The WES anti-rabbit and anti-mouse Detection Modules and the 12–230 kDa Separation Module (8 × 25) were from ProteinSimple, San Jose, CA, USA. ACE assays were performed on a ProteinSimple WES instrument.

### 2.7. Surface Plasmon Resonance (SPR) Reagents and Instrumentation

SPR reagents and Series S CM5 sensor chips were obtained from Cytiva. SPR assays were performed on a BIAcore T200.

### 2.8. Western Blot and ACE Assays 

Lysates were diluted in denaturing LDS sample load buffer and water to equal final volumes, denatured at 60 °C for 5 min, cooled and loaded onto a 4–12% polyacrylamide gradient gel followed by electrophoresis using NuPAGE MOPS Running Buffer for 1.5 h at 180–200 V. The gel was transferred with an iBLOT2 instrument to a 0.20 µm PVDF membrane for 7.5 min at 25 V. The membrane was then blocked for 0.5 h, washed with manufacturer supplied buffer, and then cut in half. One half was probed with rabbit SP464 antibody, and the other half with mouse 17B4 antibody. The membrane halves were incubated for 1 h at RT on a shaker in a 10 mL volume at a 1.0 µg/mL concentration of corresponding antibody diluted into WesternBreeze antibody diluent. The membranes were washed and incubated for 30 min at RT on a shaker with the corresponding anti-species antibody alkaline phosphatase conjugate. The membranes were washed, and antibody binding was detected with a chemiluminescent substrate according to the manufacturer’s protocol and then imaged on a FluorChem HD2 Imager. A parallel lysate set used for the Western blot was prepared and processed on the WES instrument using the manufacturer’s reagents and protocols as described below. As noted by the WES manufacturer, protein molecular weight size estimates can vary between standard Western blot and ACE methodologies.

### 2.9. ACE Antibody Titration Assays

For each assay run, HH cell lysate was mixed with 0.1× WES sample buffer reagent, and reconstituted WES 5× Fluorescent Mix reagent followed by denaturation at 95 °C for 3 min and then held on ice followed until use. Denatured sample was added to each of the designated wells of the separation module sampling plate. Dilutions of the stock rabbit SP464 and mouse 17B4 monoclonal antibodies were made to the desired concentrations using the WES detection module Antibody Dilution Buffer 2 reagent. Diluted antibody solutions were added to the designated wells of the separation module sampling plate. The remaining WES assay reagents were finally added to the plate and then processed in the WES instrument using the default running and detection parameters. 

### 2.10. ACE Fragmented Immunogen and Alanine Substituted Peptide Inhibition Assays

HH cell lysate samples were prepared and used as above for antibody titration assays. Rabbit SP464 and mouse 17B4 antibodies were diluted to 4 µg/mL in the WES detection module Antibody Dilution Buffer 2 reagent. The antibody solutions were then diluted 1:1 (vol/vol) with varying concentrations of peptide, or diluent as a no peptide control, for 1 h at RT, and 10 µL of each antibody/peptide solution was added to the designated wells of the separation module sampling plate. Samples were run in duplicates or triplicates. Plates were then processed in the WES instrument using default running and detection parameters. The IC_50_ value for each alanine-substituted peptide was determined using a non-linear fit of the log concentration of peptide inhibitor versus the combined peak areas of the soluble and full-length endogenous LAG3 bands using the four-parameter model of Prism GraphPad Prism with the bottom value constrained to zero.

### 2.11. SPR Epitope Binding Kinetics Assays

SPR epitope binding analysis was done as previously described [25,26]. Briefly, SPR kinetic analyses of peptide binding of both clones was performed on a BIAcore T200 using Series S CM5 sensor chips. The rabbit or mouse antibody capture surface was prepared with 50 ng/mL goat anti-species IgG Fc in 10 mM Acetate pH 5. Surface conditioning was performed with 50 nM of the species-specific whole molecule IgG for 120 s at 75 µL/min for five cycles. Rabbit surface regeneration was accomplished using a four step process: 15 s of 10 mM HEPES, 150 mM NaCl at 20 µL/min, 20 s of glycine pH 1.5 at 30 µL/min, and two injections of 30 s each with glycine pH 1.7 at 30 µL/min. Mouse surface regeneration was accomplished using a two-step process: two injections of glycine pH 1.7, one for 60 s at 30 µL/min and the other for 180 s at 20 µL/min. Kinetic measurements were performed at 25 °C in a solution of 10 mM HEPES, 150 mM NaCl, and 0.05% Tween. Antibody solution at 5 nM was captured for 120 s at 20 µL/min, and peptide interactions, visualized as binding curves, were measured for 400 s of association and 800 s of dissociation at 100 µL/min. Full immunogen peptide concentrations at 100, 33, 20, 11, and 3.7 nM were tested, with the 33 nM concentration measured in triplicate. Alanine substitution peptides were tested, without replicates, at 100 and 20 nM. Kinetic coefficients were calculated with BIAcore T200 Evaluation software using the 1:1 Langmuir binding model with global fit parameters and the bulk refractive index value held constant at zero. Curve fitting was done with least squares regression using Levenberg-Marquadt regression; half-life is calculated using the standard equation.

### 2.12. IHC Peptide Inhibition Assays

Cell line and tissue IHC samples were processed on a Roche Tissue Diagnostics BenchMark ULTRA automated staining platform using OptiView DAB detection chemistry (VMSI Cat# 760–700) with Hematoxylin II (790–2208) and Bluing (760–2037) counterstain. Bulk staining reagents were from Roche Tissue Diagnostics. The SP464/17B4 assay conditions consisted of 1 h of heat-based epitope retrieval on 4 µm thick sections of FFPE hepatocellular carcinoma specimens. Rabbit SP464 antibody was tested at a concentration of 49 nM (7.3 µg/mL) and mouse 17B4 antibody was tested at 263 nM (39.5 µg/mL). Primary antibody was pre-incubated with varying concentrations of peptide, for at least 60 min at 4 °C with mixing, before being incubated on-slide for 16 min at 37 °C. Each peptide was tested, in duplicate, at concentrations of 5000, 500, and 50 nM. Slides were scored by a qualified reader using the intensity of the DAB stain using a common 0 (no signal) to 3 (most intense) scale, and the percent of tumor tissue that is stained. LAG3 positive T cells typically display a punctate cytoplasmic staining pattern. Macrophages and plasmacytoid dendritic cells can also be stained with a lighter diffuse cytoplasmic pattern, however, for the purposes of this assay, that cell population is not included in the analysis. Digital images were captured on an Aperio imager at 40× magnification.

### 2.13. Chromogenic Multiplex IHC Assay

Fully automated multiplexed detection was performed on a Ventana Benchmark Ultra staining platform as described [27,28,29,30]. Detection was done using the chromogenic reagents 4-(4-dimethylaminophenylazo) benzensulfonyl (Dabsyl), carboxytetramethylrhodamine (TAMRA), and Cy5-based chromogens, available commercially in the DISCOVERY RUO Yellow kit (Cat. No. 760–239), DISCOVERY RUO Purple kit (Cat. No. 760–229), and DISCOVERY RUO Teal kit (Cat# 760–247), respectively. These kits also include the requisite enzyme-antibody conjugates. Serial tissue sections were stained with each marker and chromogen individually, and in a sequential multiplex assay. Briefly, slide mounted paraffin sections were deparaffinized and antigen retrieval was done using ULTRA Cell Conditioning 1 (950–224) incubation at 94 °C for 64 min. Staining of each biomarker was performed in sequential steps that included incubation with primary antibody at 37 °C, followed by secondary anti-species antibody conjugated to either peroxidase or alkaline phosphatase depending on whether the chromogen in sequence is a tyramide or quinone methide derivative. LAG3 (SP464) with Purple chromogen was stained first, CD8 (SP239) with Teal was stained second, and FAPα (SP325) with Yellow was stained third. Heat denaturation at 100 °C for 8 min in ULTRA Cell Conditioning 2 (950–223) was used to deactivate the antibody complex between staining cycles. Hematoxylin II (790–2208) and Bluing (760–2037) were used to counterstain. Slides were scanned at 20× magnification using a VENTANA DP200 imaging system. 

## 3. Results

### 3.1. Western Blot, IHC, and ACE Analysis of Rabbit SP464 and Mouse 17B4 Target Binding

**Figure 1:**
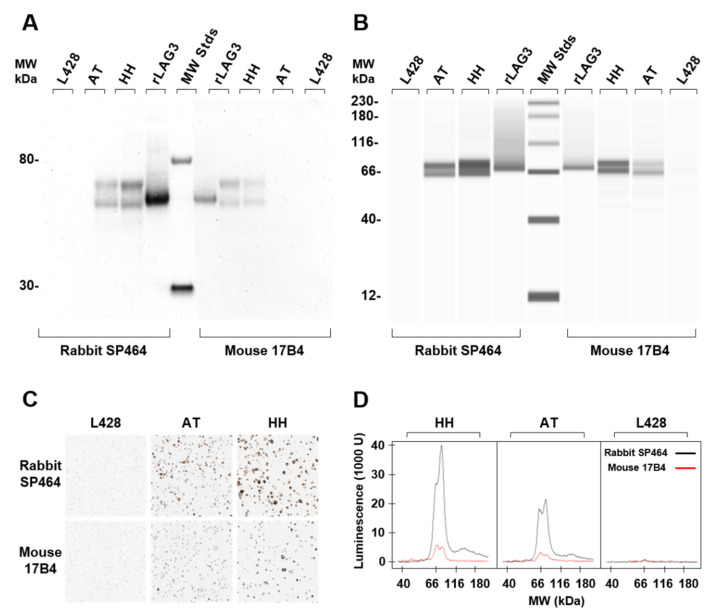
Comparison of rabbit monoclonal antibody SP464 with mouse monoclonal antibody 17B4. Western blot analysis (**A**) and ACE analysis (**B**) of recombinant LAG3 and cell line lysates probed with SP464 or 17B4. The contrast of the lanes containing recombinant LAG3 in panel B have been reduced to discern the position of the recombinant LAG3 band from the smear due to protein overload in the capillaries. Both Western blot and ACE identify two species of LAG3 protein, the full length (~58 kDa) and the soluble form (~52 kDa), in the AT and HH cell line lysates. (**C**) IHC using SP464 (upper panels) and mouse 17B4 (lower panels) to stain the cell lines L428 (negative), AT (low expresser), and HH (high expresser). (**D**) Luminescence signal intensity of cell line lysates separated by ACE and detected with SP464 (black line) and 17B4 (red line).

To determine the specificity of the rabbit SP464 and mouse 17B4 antibodies, recombinant LAG3 and protein lysates from three human cell lines were analyzed by Western blot (Figure 1A). Both antibodies detected a single band from the recombinant protein which migrates at about 53 kDa, with the SP464 signal appearing 2.5× more intense, as estimated by densitometry, compared to that of the 17B4 antibody. Two bands were observed using rabbit SP464 in the human lymphoma cell lines, one corresponding to soluble endogenous LAG3 (52 kDa) and a second higher intensity band corresponding to full length endogenous LAG3 (58 kDa). A similar banding pattern for the LAG3 recombinant protein and the three cell lines was also observed for the 17B4 antibody, however, the signal intensity for all bands was substantially less than observed for SP464. The same cell lysate preparations used for the Western blot assay were subjected to ACE analysis (Figure 1B). The corresponding banding patterns and staining intensity for each antibody recapitulated those of the Western blot (Figure 1D). FFPE cells from each cell line were stained by IHC with each antibody (Figure 1C). The staining intensities paralleled those of both the Western blot and ACE analysis. For SP464, no staining was observed in the L428 negative control cell line, moderate staining was observed for the AT cell line, and intense staining was observed for cell line HH. A similar staining pattern was observed for 17B4, but the overall staining intensity was substantially reduced. 

### 3.2. Binding Properties and Binding Kinetics of the Rabbit SP464 and Mouse 17B4 Antibodies

**Figure 2:**
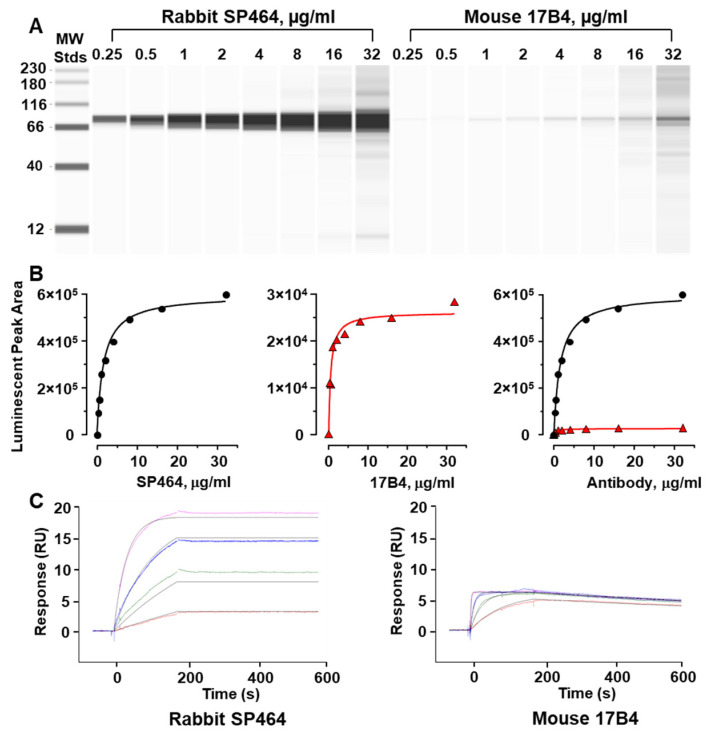
Binding characteristics of rabbit SP464 and mouse 17B4 monoclonal antibodies. (**A**) Increasing concentrations of antibody were applied to the HH cell line lysate in an ACE assay. (**B**) Curves were generated from the quantitation of luminescent signal peak areas versus antibody concentration for the rabbit SP464 (black circles) and the mouse 17B4 (red triangles). Comparison of the clones using the same y-axis is shown on the right. (**C**) Antibody binding kinetics using SPR analysis was done for SP464 (left) and 17B4 (right) using increasing concentrations of the full-length (30-mer) immunogen. Different concentrations are displayed in colors, while the global curve fits are displayed with black lines. Both plots have the same axes for time scale and response units.

To examine the binding properties of the two anti-LAG3 clones, ACE was used to probe lysate from the highest expressing cell line, HH, with varying concentrations of each antibody (Figure 2A). The signal intensity increased with increasing concentrations of each antibody, however, the signal intensity of the mouse 17B4 antibody was substantially reduced compared to that of the rabbit SP464. A plot of the concentrations versus the signal for each antibody showed a typical hyperbolic binding curve (Figure 2B). However, superposition of the curves at the same scale indicates that the maximal binding signal for SP464 is approximately 20× greater than for 17B4 (Figure 2B). 

Both the mouse 17B4 and the rabbit SP464 antibodies were created using the same 30-mer immunogen peptide sequence, “GPPAAAPGHPLAPGPHPAAPSSWGPRPRRY”. The binding kinetics of each of the two antibodies for the immunogen were examined by SPR at 25 °C (Figure 2C). The association rate constant (Ka) and dissociation rate constant (Kd) for SP464 was 2.8 × 10^5^ M^−1^ s^−1^ and 1.2 × 10^−6^ s^−1^, respectively. The Ka and Kd for 17B4 was 5.4 × 10^6^ M^−1^ s^−1^ and 6.6 × 10^−4^ s^−1^, respectively. The affinity (KD) for both SP464 and 17B4 was 4.1 × 10^−12^ M and 1.2 × 10^−10^ M, respectively. Despite a faster on-rate for the 17B4, the KD difference between the two clones is about 30×, arising from the dissociation rate being approximately 500× faster for the mouse 17B4 than the rabbit SP464 antibody. Generally, run-to-run variance can have 3× range in values, while a 10× difference is a clear delimiter. This limit was used to define significance in the criticality of residues during the binding event in subsequent analyses. 

### 3.3. Mapping of Rabbit SP464 and Mouse 17B4 Epitopes on the 30-Mer Immunogen

**Figure 3:**
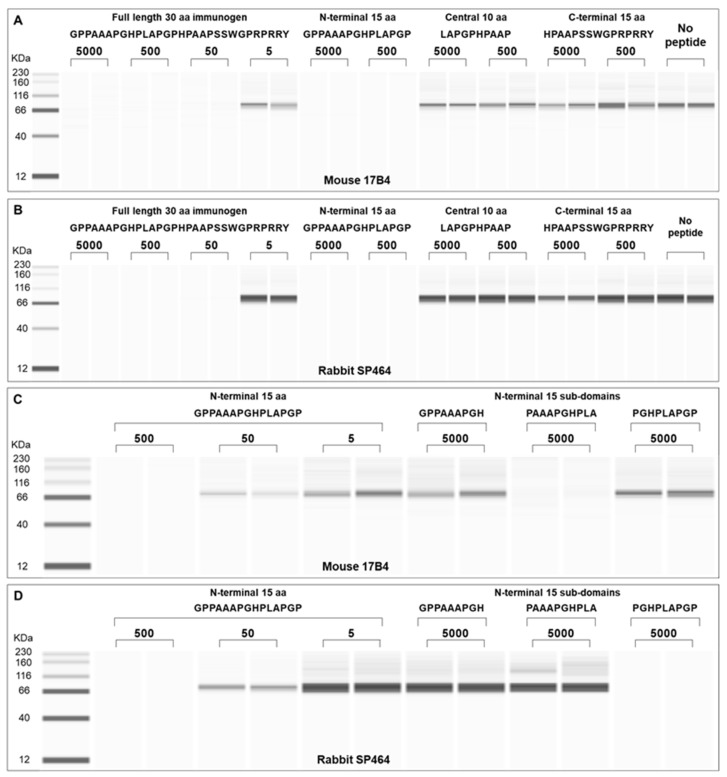
Peptide mapping of epitopes recognized by mouse 17B4 and rabbit SP464 using the ACE assay. Antibodies were incubated with the full-length immunogen or overlapping sequences derived from the *N*-terminal, central, or C-terminal region. The *N*-terminal sequence fully inhibits both 17B4 (**A**) and SP464 (**B**). Further fragmentation of the *N*-terminal sequence with overlapping sub-domain peptides is shown in (**C**) (17B4) and (**D**) (SP464). These panels identify the unique epitopes for each antibody and show complete inhibition with the center sub-domain fragment for the mouse 17B4 and the C-terminal sub-domain fragment for the rabbit SP464.

To identify the specific epitope recognized by the two antibodies, three separate peptides spanning the *N*-terminus, the center, and the C-terminus of the 30-mer immunogen were used in an ACE-based peptide inhibition assay (Figure 3). Decreasing concentrations of each synthetic peptide was incubated with each antibody and used to probe immobilized LAG3 from the HH cell lysate used above. The full-length immunogen eliminated antibody binding at concentrations greater than 50 nM for both 17B4 (Figure 3, Panel A) and SP464 (Figure 3, Panel B). The peptide consisting of the *N*-terminal 15 amino acids of the 30-mer immunogen was similarly effective in preventing antibody binding at concentrations of 5000 nM and 500 nM peptide. The peptide containing the central ten amino acids of the immunogen failed to inhibit either antibody at the same concentrations of peptide used for the *N*-terminal fragment, nor did a fragment containing the C-terminal 15 amino acids of the 30-mer immunogen inhibit either antibody. These data indicate that the epitope for both antibodies resides within the *N*-terminal 15 amino acids of the 30-mer immunogen. Further epitope mapping was done using three overlapping peptides from within this 15 amino acid sequence of the immunogen for both 17B4 (Figure 3, Panel C) and SP464 (Figure 3, Panel D). Only the central ten amino acid peptide fragment inhibited binding of the mouse 17B4, while the peptide fragment consisting of the nine C-terminal amino acids inhibited binding of the rabbit, SP464. These data localize the epitope of the mouse 17B4 antibody to the nine amino acid sequence PAAAPGHPLA, and the epitope of the rabbit SP464 antibody resides in nine amino acid sequence PGHPLAPGP. 

### 3.4. Identification of Amino Acids in the SP464 Epitope Critical for Binding to Rabbit SP464 

**Figure 4:**
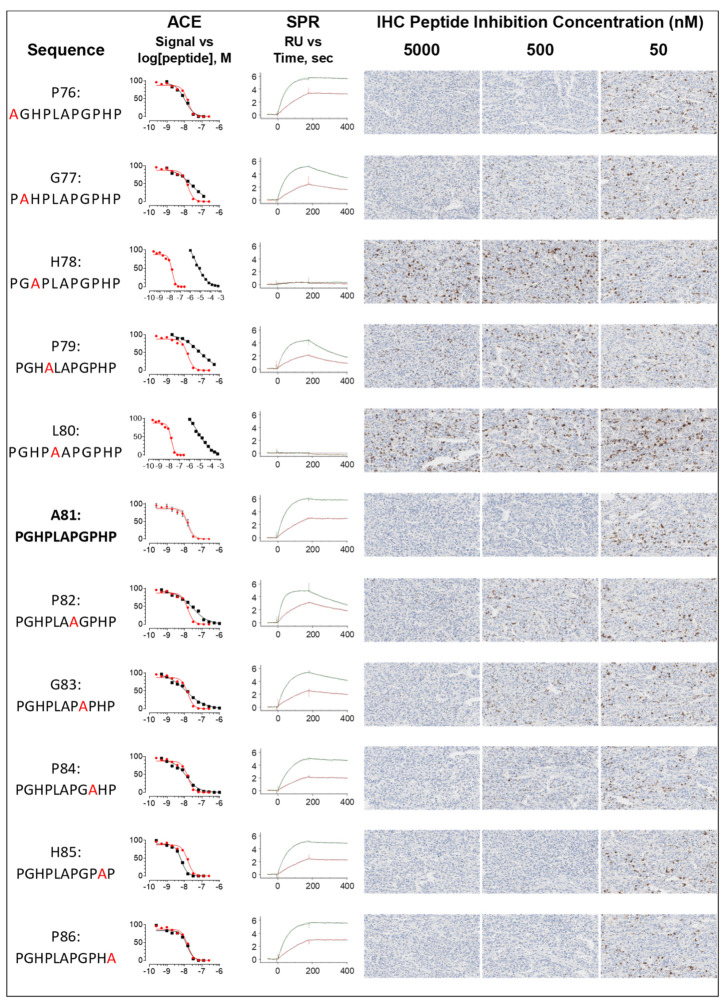
Fine epitope mapping of the SP464 target sequence by alanine substitution using ACE, SPR and IHC. Column 1 shows the substituted amino acid and its position in the full-length protein sequence, with the alanine substitution in red. Column 2 provides the ACE data dose response curves for concentrations of peptides covering several orders of magnitude. For these plots, each alanine substitution curve (black squares) is graphically compared to the wildtype curve (red circles). Column 3 contains the SPR response plots showing the signal from the two tested concentrations of peptide, 100 nM (green) and 20 nM (red). The final columns show IHC images of peptide inhibition in a tissue section of hepatocellular carcinoma at the provided concentrations. Image shown represents a 300 by 500 square micron tissue region with an apparent magnification of 20×.

To determine the contribution of each amino acid residue to antibody recognition and fully map the epitope for the new antibody SP464, ACE, SPR, and IHC were used to examine the effect of alanine substitutions at each position in the SP464 epitope. The ability of varying concentrations of each substituted peptide (Figure 4) to inhibit SP464 binding to LAG3 after pre-incubation with SP464 antibody was analyzed by all three techniques. Comparison of ACE inhibition curves (Figure 4, column 2), SPR binding curves (Figure 4, column 3), and images of the IHC peptide inhibition (Figure 4, columns 4–6) are shown for each alanine substituted peptide (Figure 4, column 1). Quantitative metrics are summarized in Table 1. 

Alanine substitution at the eighty-first amino acid (A81) serves as a positive control for unaltered function since the alanine is naturally occurring. Binding inhibition as measured by ACE demonstrates an IC_50_ of 11 nM and a Hill slope of −1.7. The SPR binding curve shows the characteristic SP464 shape, as shown in Figure 2C, with the straight-line signal during the dissociation phase, indicating that the peptide-antibody complex is very stable and not dissociating. Kinetically, the control has a 0.3 nM affinity based on an association of 1.9 × 10^5^ M^−1^ s^−1^ and a dissociation of 5.5 × 10^−5^ s^−1^, resulting in a calculated half-life of 211 min. Stability of the complex was further demonstrated, as shown by the IHC images. In the absence of peptide, 50% of the tissue stains at the highest DAB intensity score of 3, as shown in Figure 4 and Table 1, for the L80 alanine substitution peptide. At near molar equivalent concentrations of peptide and antibody, the IHC signal is only modestly reduced in intensity, while higher concentrations of the peptide (5000 nM to 500 nM) are required to inhibit 50 nM of antibody binding to the target tissue. 

Based on these values, cut-off distinctions between the wild type control peptide and the alanine substituted peptides were defined as follows: Critical residues are defined by the lack of an interaction (shown graphically) while KD values 10× greater than the A81 (control) peptide KD, 0.3 nM, are considered contributory. For IHC assays, tissue heterogeneity is a major factor of variability, and can contribute as much as 10% difference in percent coverage. Therefore, for this assay, an increase of 1–3% over baseline is considered not significant. Signal intensity changes of more than 0.5 from the highest score (3), are considered significant indicating partial inhibition, with a signal intensity of zero indicative of complete inhibition. Critical residues are those defined by a lack of inhibition at the highest peptide concentration. Full to partial inhibition at a peptide concentration of 500 nM is considered contributory. Two values derived from ACE analysis, the IC_50_ and the Hill slope, were used in tandem to identify critical residues. An IC_50_ greater than 10×, compared to the wildtype, is deemed critical. IC_50_ values that are 2× or greater are considered contributory. Hill slope values 50% lower than the Hill slope of the wild type peptide, −1.7, are considered contributory. Experimental variance for ACE analysis has not been previously established, however small perturbations in binding characteristics can theoretically be used to delineate the minimum epitope. 

All three techniques, ACE, SPR, and IHC demonstrate that amino acids H78 and L80 are critical residues, for SP464 epitope binding. As shown in Figure 4, ACE inhibition curves for alanine replacement of H78 and L80 demonstrated IC_50_ values of 3600 nM and 6000 nM, respectively, a shift of more than 300× and 500×, when compared to the wildtype peptide. The SPR binding curves show no interaction between the antibody and peptide. IHC images show no inhibition of DAB signal at all concentrations; stain intensity and percent coverage are commensurate with peptide-free IHC staining. 

Amino acids that contribute to SP464 binding but are not critical include G77, P79, P82 and G83. The ACE inhibition assay using the alanine substitution peptides G77, P79, and P82 produced IC_50_ values that are more than 2× greater than the control peptide, but do not reach 10×. There is flattening of the H78, P79, and L80 peptide binding curves, resulting in a Hill slope value that approaches zero (greater than −1.7). The SPR binding curves of these peptides, and G83, show greater complex dissociation, compared to the control curves, with a dissociation 100× higher, representing faster dissociation. The resulting affinities are more than 10× greater than the control KD and the half-lives were reduced to times between 2 to 10 min. Similarly, the IHC images for the G77 substitution show DAB signal partially restored at both 500 and 50 nM concentrations, with lower signal intensity at 500 nM. The IHC images for P79, P82, and G83 substitutions also show partial inhibition of the DAB signal intensity at the highest concentration of peptide (5 µM). These data suggest that G77, P79, P82, and G83 contribute to the antibody affinity, but are not critical for epitope recognition. 

The ACE metrics, IC_50_ and Hill slope for P76, P84, H85, and P86, are nearly equivalent between these peptides and the control sequence. SPR binding curves closely resembled the control curves and the affinity for these peptide substitutions is less than 10× greater than the wild type KD. IHC images of these peptides used in the inhibition assay show DAB signal only at the lowest peptide concentration with a signal intensity representative of the peptide-free intensity. These data suggest that amino acids P76, P84, H85, and P86 may make minor contributions to antibody binding, however the nature of the interaction is unknown. The overall agreement between each of the techniques used for assessing the binding contributions of each of the amino acids within the SP464 epitope as determined by ACE, SPR, and IHC is good, suggesting overall that the minimal epitope likely consists of the sequence GHPLAPG. The comparative results are summarized in Table 2.

### 3.5. Application of SP464 in a Chromogenic Multiplex Assay

**Figure 5:**
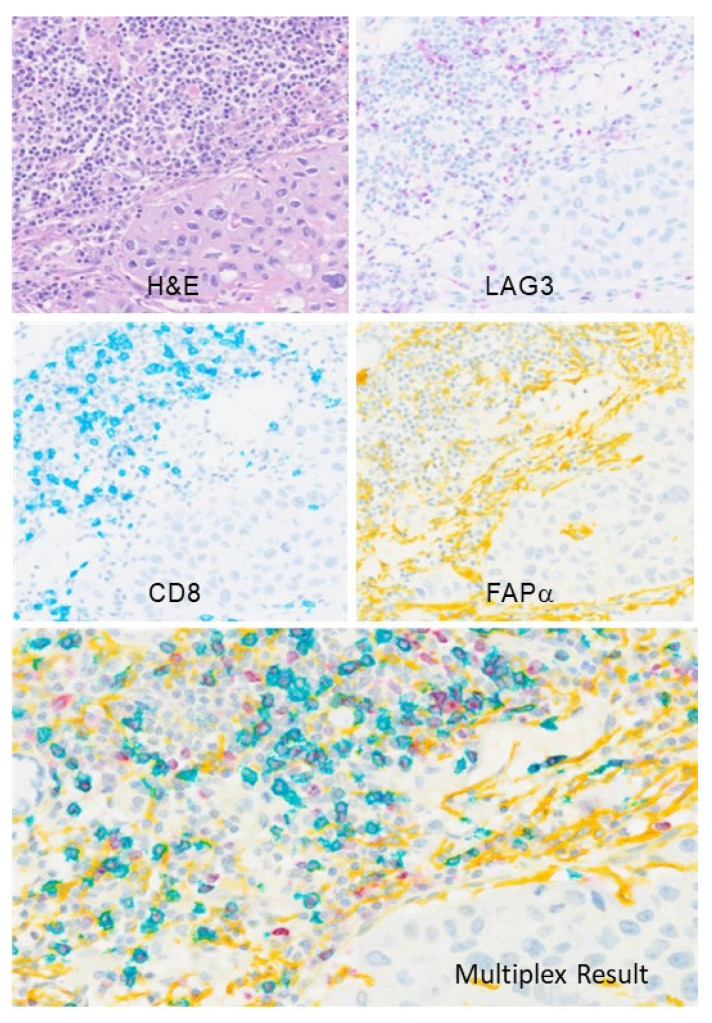
Multiplex IHC of serial NSCLC tissue sections stained using traditional hematoxylin and eosin (H&E), anti-LAG3 clone SP464 (seen in purple), anti-CD8 (SP239) (seen in teal), and anti-fibroblast activation protein, alpha (FAPα, SP325) (seen in yellow). Chromogenic multiplex allows the visualization of all three antibodies on a single tissue section, as shown in the “Multiplex Result” panel.

To investigate the potential of SP464 as an IVD reagent, FFPE sections from a non-small cell lung carcinoma (NSCLC) tissue were stained in a chromogenic multiplex IHC assay. Serial sections were stained using the anti-LAG3 clone SP464 (purple), anti-CD8 (SP239) (teal), and anti-Fibroblast activation protein, alpha (FAPα, SP325) (yellow) or sequentially with all three antibodies (Figure 5). Anti-CD8, identifies T cells, and anti-FAPα, identifies tumor stroma. Activated T cells can be seen by co-expression of LAG3 (purple) primarily in a punctate cytoplasmic pattern within membrane stained CD8 positive T cells (teal) or as a blended blue-violet hue. Cells stained with LAG3 only (purple) are most likely tissue resident macrophages or plasmacytoid dendritic cells. 

## 4. Discussion

The advent of precision medicine and the introduction of the drug-diagnostic co-development strategy has driven the need for reliable IVDRs that identify predictive biomarkers [31]. Biological reagents used for diagnostic and therapeutic purposes are often defined functionally and lack rigorous identification of the epitope target. Herein, we demonstrate that a combinatorial approach utilizing three powerful and orthogonal methods, ACE, SPR, and IHC can be used to not only elucidate the target epitope sequence, but can additionally characterize the specificity, affinity, and stability of an antibody-epitope interaction with commensurate accuracy. 

Antibody characterization and target identification typically relies on Western blot analysis, in which the antibody is required to bind to a denatured epitope in the context of a full-length protein. Automated capillary electrophoresis, the ACE assay, is based on the Western blot principle; however, it utilizes micro volumes of sample separated in a gel coated capillary tube. We used a novel ACE approach to map the epitopes of the 17B4 and SP464 antibodies within the 30-mer immunogen peptide sequence using competitive peptide inhibition assays with peptide sequence fragments derived from the immunogen. Further analysis identified both the critical and contributory residues for the SP464 antibody. Comparing across all three techniques, the substitution of the H78 and L80 residues with alanine results in complete loss of function, demonstrating that these amino acids are critical for the peptide-antibody complex. Furthermore, our approach revealed that the rabbit SP464 antibody epitope lies adjacent to and partially overlaps with that of the mouse 17B4 epitope. 

To validate the sensitivity and epitope specificity identified by ACE, we used peptide binding kinetics by SPR, and functional assays by traditional IHC. The underlying technology of SPR enables the study of binding events with picomolar sensitivity, as peptide binding events on the chip surface alters the signal response. The IHC assay requires the antibody to recognize the protein in its native context within the cellular milieu and provides functional significance to the use of fine epitope mapping in IVDR development. For instance, comparisons of the SPR-based signal intensities show that the maximal binding signals were obtained for SP464, which exceeded those of 17B4 by approximately 20×, therefore suggesting that within an IVDR application, a wider range of LAG3 expression would be detected by SP464, than is possible with the mouse 17B4.

Immunotherapy with immune-checkpoint inhibitors has revolutionized cancer therapy. Companion and complementary diagnostic assays for PD-L1 have shown an association between tumor cell expression of PD-L1 and the efficacy of immune checkpoint inhibitors, however a subset of patients fail to respond in spite of high tumoral PD-L1 expression. One mechanism that may contribute to checkpoint inhibitor resistance is compromised T cell function due to inhibitory signals including LAG3 receptor activation. The recent FDA approval of the anti-PD1 and anti-LAG3 combination therapy highlights the need for greater precision in diagnostic assays to identify patients that may benefit from combination therapies. The RELATIVITY-047 clinical trial reported retrospective assessment of LAG3 expression in enrolled patients using a laboratory-developed test based on the 17B4 antibody [4,32]. Although the median progression-free survival was longer for patients with expression of 1% or greater, benefit was also seen when LAG3 was not detected, suggesting that an assay employing an anti-LAG3 monoclonal antibody with greater sensitivity and specificity could improve patient selection. Multiplexing assays utilizing highly sensitive and specific anti-LAG3 antibodies combined with additional biomarker specific antibodies, such as CD8, may prove useful for further assessing the relative biomarker distributions in various tumor types and thereby facilitate prognostic assessments [33,34].

Currently a number of mouse and rabbit anti-LAG3 monoclonal antibodies are commercially available, however, with the exception of the widely used mouse 17B4 clone, the epitopes and binding parameters of these other antibodies are either unknown or proprietary. In this study comparing the properties of the rabbit SP464 and mouse 17B4 clones, we have shown that a combination of ACE, SPR, and IHC constitutes an extremely powerful pre-clinical approach for epitope identification and for assessing and comparing the binding parameters of antibodies in development for clinical applications. This work establishes the technique and mathematical metrics in the novel usage of ACE for finely mapping a minimum epitope contained within a larger immunogen and identifying critical and contributory roles of specific amino acids within the epitope. The value of fine epitope mapping is the identification of the precise location of epitope boundaries and the amino acids that are critical for the antibody target epitope interaction. Knowledge of the precise sequence of a monoclonal antibody target epitope is critical for assessing the extent of target specificity. Peptide sequences in non-target proteins having either full or partial homology to the epitope of the target protein may detract from or even nullify the clinical utility of an antibody. This aids in the identification of cross-reactive sites in other proteins. Furthermore, by identifying critical contacts that are essential for binding, potentially cross-reactive sequences, lacking one or more of the critical contact sites, can be excluded from a BLASTP protein sequence alignment analysis. Our approach enables the comparative characterization of antibody binding parameters, localizes the target epitope sequence within an immunogen, identifies amino acids that are critical and contributory to antibody binding, and facilitates bioinformatic identification of potential off-target sequences. 

## Figures and Tables

**Table 1:** Effect of alanine substitution on the SP464 epitope binding parameters as measured by ACE, SPR, and IHC.

	ACE		SPR				IHC Score (% Positive)		
Peptide	IC_50_, nM	Hill Slope	Ka, 1/Ms	Kd, 1/s	KD, nM	Half-Life, min	5000 nM	500 nM	50 nM
**P76**: AGHPLAPGPHP	10	−1.7	2.4 ×10^5^	1.3 ×10^−4^	0.6	88	0	0	3 (30%)
**G77**: PAHPLAPGPHP	26	−0.95	1.8 ×10^5^	1.9 ×10^−3^	10	6	0	2 (20%)	3 (30%)
**H78**: PGAPLAPGPHP	3600	−0.81	NA	NA	No Binding	NA	3 (50%)	3 (50%)	3 (50%)
**P79**: PGHALAPGPHP	74	−0.81	2.3 ×10^5^	4.6 ×10^−3^	20	2	1 (10%)	3 (30%)	3 (50%)
**L80**: PGHPAAPGPHP	6000	−0.65	NA	NA	No Binding	NA	3 (50%)	3 (50%)	3 (50%)
**A81: PGHPLAPGPHP**	**11**	**−1.7**	**1.9 ×10^5^**	**5.5 ×10^−5^**	**0.3**	**211**	**0**	**0**	**2 (50%)**
**P82**: PGHPLAAGPHP	28	−0.97	3.7 ×10^5^	2.5 ×10^−3^	6.9	5	1 (2%)	2 (50%)	3 (50%)
**G83**: PGHPLAPAPHP	13	−0.90	1.7 ×10^5^	1.1 ×10^−3^	6.5	10	1 (1%)	2 (20%)	2 (50%)
**P84**: PGHPLAPGAHP	13	−1.2	1.3 ×10^5^	2.9 ×10^−4^	2	40	0	1 (3%)	2 (50%)
**H85**: PGHPLAPGPAP	6.5	−2.4	1.6 ×10^5^	1.5 ×10^−4^	0.9	78	0	1 (1%)	2 (50%)
**P86**: PGHPLAPGPHA	14	−2.4	2.0 ×10^5^	6.2 ×10^−5^	0.3	188	0	0	2 (50%)

The first column contains the name of the peptide in bold and the location of the alanine substitution in red. A81 is completely in bold as it the wildtype and has no substitution. Kinetic constants determined by SPR are provided describing association rate constant (Ka), dissociation rate constant (Kd), and affinity (KD). IHC score is provided per peptide inhibition concentration along with the percent of tumor that is positive in parentheses.

**Table 2:** Summary of critical (red background), contributory (blue background), and residues without effect (white background) on the SP464 antibody-peptide binding event as determined by ACE, SPR, and IHC.

Amino Acid Designation:		P76	G77	H78	P79	L80	A81	P82	G83	P84	H85	P86
ACE	IC_50_	P	G	H	P	L	A	P	G	P	H	P
	Hill Slope	P	G	H	P	L	A	P	G	P	H	P
SPR		P	G	H	P	L	A	P	G	P	H	P
IHC		P	G	H	P	L	A	P	G	P	H	P

## Data Availability

All data are contained within the article.
